# Surface Functionalization Studies in the Development of Nanohole Plasmonic Sensors

**DOI:** 10.3390/s26113434

**Published:** 2026-05-29

**Authors:** Sezin Sayin, Kristen L. Steffens, Kurt D. Benkstein, Mona Zaghloul, Steve Semancik

**Affiliations:** 1 Department of Electrical and Computer Engineering, School of Engineering and Applied Science, The George Washington University, Washington, DC 20052, USA; zaghloul@gwu.edu; 2 Biomolecular Measurement Division, National Institute of Standards and Technology (NIST), Gaithersburg, MD 20899, USA; kristen.steffens@nist.gov (K.L.S.); kurt.benkstein@nist.gov (K.D.B.); stephen.semancik@nist.gov (S.S.)

**Keywords:** biomedical diagnostics, biosensor, liquid-phase diagnostics, localized surface plasmon resonance, nanosensors, plasmonic sensor, surface functionalization, x-ray photoelectron spectroscopy, XPS

## Abstract

Localized surface plasmon resonance (LSPR) is an optical phenomenon that occurs when light interacts with free electrons on the surface of metallic thin films, producing intensified electromagnetic fields at specific sites, often called “hot spots”. LSPR-based sensing technologies respond to chemical and associated optical interfacial changes. Inherent advantages include enhanced sensitivity, compact size, low production cost, and strong potential for integration into portable, point-of-care diagnostic systems. This study focuses on a detailed investigation into the surface functionalization of localized surface plasmon resonance (LSPR)-based nanohole array (NHA) sensors for biomedical applications. Gold-coated NHA surfaces were functionalized using polyethylene glycol (PEG) self-assembled monolayers (SAMs), enabling specific attachment of biomolecular species. As a proof-of-concept, bovine serum albumin (BSA) and SARS-CoV-2 nanobody proteins were successfully immobilized on the PEGylated surfaces. Individual steps of surface modification including PEGylation, protein immobilization and nanobody immobilization were validated through a dual-method approach which combined measurement of LSPR optical spectral shifts and x-ray photoelectron spectroscopy (XPS) chemical analyses. Reproducibility was assessed across multiple sensors and repeated trials, confirming the repeatability of each functionalization and binding process. The sensor system, consisting of NHA-based plasmonic platform, microfluidics, and a portable optical spectrometer, exhibits the capability for reliable and sensitive, label-free detection of biomolecular targets, including viral antigens, in liquid-phase environments.

## 1. Introduction

Localized surface plasmon resonance (LSPR), like surface plasmon resonance (SPR), arises from localized surface plasmons (LSPs), which are collective oscillations of conduction electrons confined to metallic nanostructures and excited by incident light. This interaction leads to strong surface charge polarization and enhanced electromagnetic fields, significantly amplifying optical signals resulting from binding-induced interfacial optical property changes for high-sensitivity sensing applications [1,2,3]. LSPR-based sensors enable real-time, label-free detection of molecular interactions, offering insights into binding specificity, kinetics, and affinity, and are widely applied in fields such as drug discovery, antibody analysis, proteomics, immunogenicity studies, and food safety, due to their ability to detect trace amounts of analytes near the sensor surface [4].

Integrating LSPR-based sensors with microfluidics enables the development of compact, automated sensor systems ideal for biological and chemical analysis of small, controlled liquid samples [5,6,7,8]. This significantly enhances sensor performance by introducing advantages of microfluidics in liquid-phase applications, such as minimal sample volumes and reduced diffusion time and therefore sensor response time. Additionally, including microfluidics ensures greater reproducibility and precise control over liquid sample amount, making it a powerful platform for efficient, high-sensitivity sensing [9]. Semwal et al. fabricated a compact, label-free LSPR sensor using 80 nm gold nanoparticles integrated with a microfluidic chamber for stable, real-time detection of single-stranded DNA. Their platform showed high sensitivity, ≈90 nm/refractive index unit (RIU), good repeatability, and a detection limit of 0.75 nmol/L without signal amplification [10]. In another study, using SPR, Chuang et al. showed label-free detection of the LMP1 gene using a microfluidic device with a gold-capped nanowire array. Their system offers strong specificity, cost-effectiveness, and reproducibility, showing promise for compact, rapid DNA detection systems [11]. Minopoli et al. compiled and reviewed recent studies on plasmonic sensors based on nanostructures. They reported the sensitivity based on LSPR devices ranges from (100 to 1600) nm/RIU [12]. Jiang et al. studied plasmonic nano-arrays for ultrasensitive biosensing and summarized a range of (480 to 600) nm/RIU sensitivity specific to nanohole structures [13].

To enable the use of such sensor platforms for biomedical diagnostics, it is essential to capture biomolecular target species near the active surface of sensor using specific receptors. However, these receptors cannot be directly attached to the gold (Au) surface, making surface functionalization a critical step in sensor fabrication. A self-assembled monolayer (SAM) technique is employed to achieve this functionalization, with polyethylene glycol (PEG) used to create a layer of exposed carboxylic acid groups on the Au surface [14,15,16,17]. This process significantly enhances the specific binding of proteins near the sensor surface. The SAM method, utilizing methyl-PEG_4_-thiol (MT(PEG)_4_) and carboxy-PEG_12_-thiol (CT(PEG)_12_), forms a hydrophilic, methyl ether-terminated PEG layer with periodically exposed carboxylic acid groups. These exposed groups are crucial for coupling affinity ligands to ensure effective receptor immobilization. This approach improves the sensor’s ability to capture and detect biomolecular targets for diagnostic applications [18].

For PEGylation via the SAM method, as well as the next interfacial biochemical steps involving the attachment of probes, receptors and targets to the sensor surface (relating to biomedical diagnostics for disease-related proteins), we initially established the LSPR spectral shift responses for each of the biochemical modifications. Bovine serum albumin (BSA) was used first as a model protein exposed to the PEGylated surface. BSA has a mass of 66.5 kDa and was chosen due to its widespread use in biomedical applications, low cost, ease of preparation, and ability to enhance assay signals. Furthermore, BSA is also used for treating sensor surfaces to minimize non-specific binding in sensor systems [19,20,21]. Similarly, and separately, we also examined the PEG-based attachment of a SARS-CoV-2 nanobody receptor which can enable COVID-19 target detection [22,23,24]. Nanobodies are smaller in size (≈15 kDa) than antibodies, highly soluble, stable, and have low steric hindrance to reach targets [25]. These properties make them ideal for surface functionalization in compact, surface-sensitive biosensors.

While LSPR should optically respond to PEG and protein attachment in a consistent manner, it is important to further verify the nature of those responses to binding processes via an additional surface analytical method, particularly when working with a novel sensor platform in which the nanoholes are fully etched through the substrate [26]. X-ray photoelectron spectroscopy (XPS) is a spectroscopic chemical analysis technique, wherein x-rays interact with a sample surface, causing surface atoms from roughly the top 10 nm to eject inner core electrons with kinetic energies which are then related back to the specific binding energy of the electron, forming a spectrum which indicates not only the chemical composition of the surface, but also information about the chemical bonding of the atoms on the surface. As modifications are made to the surface, introducing, for instance, more carbon bound to oxygen in PEG or the presence of nitrogen in proteins, XPS can be used to detect the presence of these bound layers, making it a valuable complementary technique to LSPR in this work [27,28].

Building on the previously published design and fabrication of Au coated NHA-based LSPR sensors integrated into PDMS wells. This study focused on expanding their utility for reliable liquid-phase biomedical diagnostics through careful stepwise analyses of the device interfacial processing involving PEG SAM formation followed by modification with a protein, either BSA or the SARS nanobody. Each stage of the surface functionalization was thoroughly verified using the mentioned complementary dual-method strategy employing LSPR optical spectroscopy in conjunction with XPS. The integration of XPS as a complementary technique to LSPR was crucial for verifying the chemical composition and binding states of surface layers, thereby confirming the reliability and specificity of the detection processes. This dual-validation approach not only strengthens the platform’s diagnostic credibility but also explains its potential for high-sensitivity, label-free detection in real-world biomedical and point-of-care applications. The novelty of this study lies in its evaluation of SAM and specific receptor immobilization on sensor surfaces using complementary optical spectroscopy and XPS analyses, to show the reliability of each surface modification stepin support of future biosensing studies with the platform.

## 2. Materials and Methods

### 2.1. Materials

Sylgard 184 Silicone Elastomer Base (Dow, Midland, MI, USA) and Sylgard 184 Elastomer Curing Agent (Dow) were used for PDMS well fabrication. Methyl-PEG-Thiol Compound (MT(PEG)_4_) (Thermo Fisher Scientific, Waltham, MA, USA), Carboxy-PEG-Thiol Compound (CT(PEG)_12_) (Thermo Fisher Scientific, USA), Dimethyl sulfoxide (DMSO) (Millipore, Burlington, MA, USA), and Phosphate-buffered saline (PBS) powder packets (Sigma-Aldrich, Burlington, MA, USA) were used for the PEG self-assembly monolayer. Bovine Albumin, Fraction V (BSA) (MP Biomedicals, Solon, OH, USA) and SARS-CoV-2 Spike RBD Recombinant Nanobody (CusaBio, Houston, TX, USA) were utilized for surface functionalization.

### 2.2. NHA and PDMS Well Fabrication

NHA devices with features of 200 nm diameter and 400 nm period were fabricated as described in a previous study [29], beginning with the deposition of a 100 nm silicon nitride layer onto a 500 µm silicon substrate via low-pressure chemical vapor deposition (LPCVD). The patterning was done using electron-beam lithography (EBL) and reactive ion etching (RIE). Each NHA platform consisted of up to four identical NPA sensing sectors with size of 300 µm by 300 µm, so that separate measurements could be performed on multiple sensors within a single device platform. A membrane window was then created on the backside using a mask aligner and RIE. Finally, a 5 nm titanium layer and a 75 nm gold layer were deposited onto the surface using an electron-beam evaporator. In this study, to isolate and evaluate the reproducibility of surface functionalization procedures, the geometry and gold thickness were held constant at these values [29].

As previously described [29], the PDMS mold for the device was fabricated using a polymer solution casting technique. A computer numerical control (CNC) milling machine was used to create the mold from a 0.64 cm thick PMMA stock.

**Figure 1:**
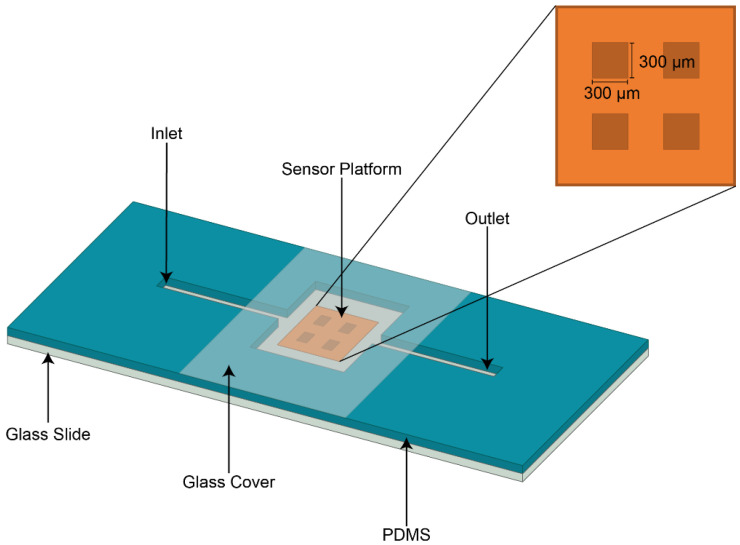
Nanohole sensor schematic with microfluidics showing liquid inlet and outlet channels and the well, which houses the plasmonic platform with four NHA-sectors.

The PDMS fabrication involved mixing base and curing agents (10:1 ratio), followed by vigorous blending and air bubble removal. The mixture was cured in an oven at 80 °C for 2 h. Once solidified, the PDMS was bonded to the glass slide using an oxygen plasma bonding technique, with a 0.25 cm gap for liquid flow through the sensor, as shown in Figure 1. The inlet–outlet channels have a length of 2.8 cm and the opening for sensor platform on the PDMS well is 0.62 cm long, 0.43 cm wide and 0.25 cm thick.

The refractive index sensitivity of this NHA-based plasmonic/microfluidic sensing platform was determined using measurements made on water–ethanol mixtures. These measurements and the refractive indices for varied ratio water–ethanol samples (see Table S1 and Figure S1) [29,30] produced a linear-fit sensitivity of 425 ± 46 nm/RIU for the studied NHA.

### 2.3. PEGylation Using the SAM Technique

The PEGylation steps for the SAM formation (see Figure S2 for surface modification chemistry) involve stock solution preparation, PEG solution preparation and coating of PEG onto surfaces, as detailed in Figure S3.

Stock solution preparation: 100 mg MT(PEG)_4_ was dissolved in 1.5 mL dimethyl sulfoxide (DMSO) (300 mmol/L). A total of 100 mg CT(PEG)_12_ was dissolved in 1 mL DMSO (150 mmol/L). Stock solutions were each mixed in a vial with a PTFE/silicone septum and stored under nitrogen at −20 °C. PBS was dissolved in 500 mL DI water. The pH was checked with a pH meter and adjusted to pH 7.2 by using 1 M NaOH and HCl solutions as necessary.

PEG solutions preparation: A total of 100 μL of MT(PEG)_4_ stock solution was transferred via pipette to a vial containing 500 μL DMSO. Then, 60 μL of this diluted MT(PEG)_4_ solution was mixed with 240 μL of PBS stock solution.

Similarly, 200 μL of CT(PEG)_12_ stock solution was transferred via pipette to a vial containing 400 μL DMSO. Then, 60 μL of this diluted CT(PEG)_12_ solution was mixed with 240 μL of PBS stock solution. Next, the two PEG solutions were combined by mixing 300 μL of the resultant MT(PEG)_4_ solution with 100 μL of the resultant CT(PEG)_12_ solution. Finally, the concentrations in the combined sample were 7.5 mmol/L MT(PEG)_4_ and 2.5 mmol/L CT(PEG)_12_.

PEG coating onto the surface: The NHA-based plasmonic sensor platform to be coated was placed in the PDMS well and 200 μL of mixed PEG solution was deposited onto the sensor surface via pipette. The sample was allowed to react for 2 h at room temperature and then washed, first with PBS and then DI water, before being dried under ambient conditions. All sensor sectors within a single platform simultaneously received the same treatment.

### 2.4. BSA and SARS-CoV-2 Nanobody Immobilization on the NHA-Based Sensor Platforms

In this work, prior to SARS-CoV-2 nanobody immobilization, BSA was used as a model proof-of-concept protein to demonstrate protein immobilization on PEGylated sensor platforms, and to verify the plasmonic response to biomolecular attachment. The amount of BSA employed in this study was 1 mg/mL in PBS solution, which is consistent with the typical literature range [22,23,24]. BSA was prepared using a normal PBS packet dissolved in 1L DI water with 200 ppm NaN_3_ as a preservative and then filtering with a 0.22 μm PES (polyethersulfone) filter prior to adding the BSA and then transferring to a small glass autosampler vial.

The number of individual nanobodies (15 kDa) immobilized is assumed to be higher than for BSA as BSA is a bigger molecule with a molecular weight of 66.5 kDa. The attachment procedure employed was essentially the same for both BSA and nanobody attachment.

The calculations for dosing were made based on example diagnostic applications with a SARS-CoV-2 nanobody of 15 kDa molecular weight. BSA and SARS-CoV-2 nanobody attachment procedure steps include the preparation of 20 mg/L solution of protein by diluting 5 μL 1 mg/mL solution in 250 μL PBS (20 mg/L), and application of 200 μL of solution to the sensor platform surface (equivalently to all sectors) in the PDMS well by micropipette. The sample was allowed to react for 2 h at room temperature. The resultant sensor surface was washed with PBS stock solution.

**Figure 2:**
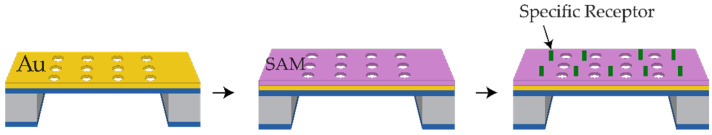
Schematic of surface functionalization steps including self-assembly with PEGylation, and specific receptor binding.

Figure 2 schematically represents the surface functionalization steps involving PEGylation self-assembly (see also Figure S2) and nanobody immobilization on the Au surface, specifically for applicability to SARS-CoV-2 detection (but with broader biomedical contexts when other aptamer or nanobody receptors would be introduced). These individual steps were characterized by both XPS and optical measurements to ensure viable functionalization processing toward a future sensor array for SARS-CoV-2.

### 2.5. Spectrometer Setup

The plasmonic sensing platform with NHA structures, described previously [29], enables the generation of localized surface plasmon resonance (LSPR) behavior when visible light interacts with the NHA’s subwavelength features (dimensions ≈ 200 nm). To obtain optical spectra from the plasmonic sensor platforms, a reflectance-based bright-field spectrometer configuration was utilized. The system employed a broadband visible light source (Motic MLC-150C, Universal City, TX, USA) to illuminate the sensor surfaces through a microscope (Motic PSM-1000, Universal City, TX, USA). The reflected light was captured by a spectrometer (Thorlabs CCS 200, Newton, NJ, USA) on a probe station (Signatone S-1160, Gilroy, CA, USA) via an optical fiber. Prior to conducting optical spectrometer measurements, the device was calibrated to remove background signals from both the gold surface and the broadband light source. Data from 1000 spectra were averaged and subsequently normalized to account for the light source and dark noise according to the following equation [31].

$$I_{\mathrm{sample}}=\frac{I_{\mathrm{analyte}}\;-\;I_{\mathrm{dark}}}{I_{\mathrm{light}}\;-\;I_{\mathrm{dark}}}$$

The light source was allowed to warm up for one hour, and parameters such as lamp power, sweep range, measurement mode, and magnification were optimized accordingly. Data processing and analysis, including fitting, background subtraction, and resonance peak determination [29,31], were performed using OriginPro 2020b software (Northampton, MA, USA). As the resonance peak analysis employed comparative measurements of resonance shifts after each step of surface functionalization, a consistent data fitting protocol was used for all spectra. Peaks were modeled as a 9th order polynomial which gave the optimal data fitting with minimal order for full peak profile. The selection of 9th order polynomial was based on the empirical evaluation of data fitting performance across the range of polynomial orders. Lower order polynomials were insufficient to include variations, which resulted in systematic deviations. Therefore, a 9th order polynomial model was selected as optimal with respect to data fitting accuracy and ease of implementation.

Spectrometer measurements were performed individually for each of three NHA sectors within the multi-sector platform by appropriate microscope stage translation, with three LSPR measurement trials acquired on each sector for the Au/NHA, PEG/Au/NHA, BSA/PEG/Au/NHA, and SARS-CoV-2 nanobody/PEG/Au/NHA samples in PDMS wells with PBS solution. Reported mean peak positions and standard deviations are all based upon *n* = 3 measurements. These repetitions were used to assess measurement consistency and reproducibility.

### 2.6. XPS Setup

XPS analyses were performed using a Kratos Analytical Axis-Ultra DLD X-ray Photoelectron Spectrometer (Kratos Analytical, Inc., Nanuet, NY, USA) with a monochromated Al Kα source (λ = 1486.7 eV, 150 W) and a nominal analysis spot size of diameter 110 μm for the patterned NHA sectors, detected normal to the surface. Spectra were collected for each NHA sector, optimizing the position using signal intensity, which decreased over the arrays due to the structured surface. The sector area is 300 µm × 300 µm and four sectors are separated by 400 µm. An additional spot outside the array area was analyzed as a control. Low-resolution survey spectra (160 eV pass energy, 0.5 eV step size) were obtained at each position, averaging three scans for each survey spectrum. In addition, high-resolution spectra (40 eV pass energy, 0.1 eV step size) were measured for the Au 4f, C 1s, O 1s, N 1s, Cl 2p, F 1s, Si 2p and S 2p electron core levels, averaging 5 to 10 scans per spectrum. XPS curve-fitting and analysis was performed using CasaXPS software (v. 2.3.24PR1.0, Casa Software Ltd., Devon, UK). For all elements except for Au, a linear background was subtracted prior to fitting peaks with a 70% Gaussian/30% Lorentzian peak shape. For Au, a Shirley type background was subtracted, and peaks were fit using a 20% Gaussian/80% Lorentzian peak shape. The binding energy scale was calibrated to the Au 4f_7/2_ peak at 84.0 eV. In addition to the NHA sensor samples that were studied with optical spectroscopy (LSPR) for all conditions of functionalization, Au coated Si macrosamples, both with and without PEGylation, were characterized using XPS. For these macrosamples, as well as the NHA background control spots, the XPS analysis area was 700 µm × 300 µm. For all XPS samples, multiple spots (2 to 6) were analyzed, and representative spectra are presented in the figures. In all cases, the background was subtracted prior to plotting, unless indicated otherwise. The naming convention for various samples examined can be found in Table 1.

## 3. Results and Discussion

### 3.1. XPS Results

#### 3.1.1. Surface Functionalization via PEG Self-Assembly

**Figure 3:**
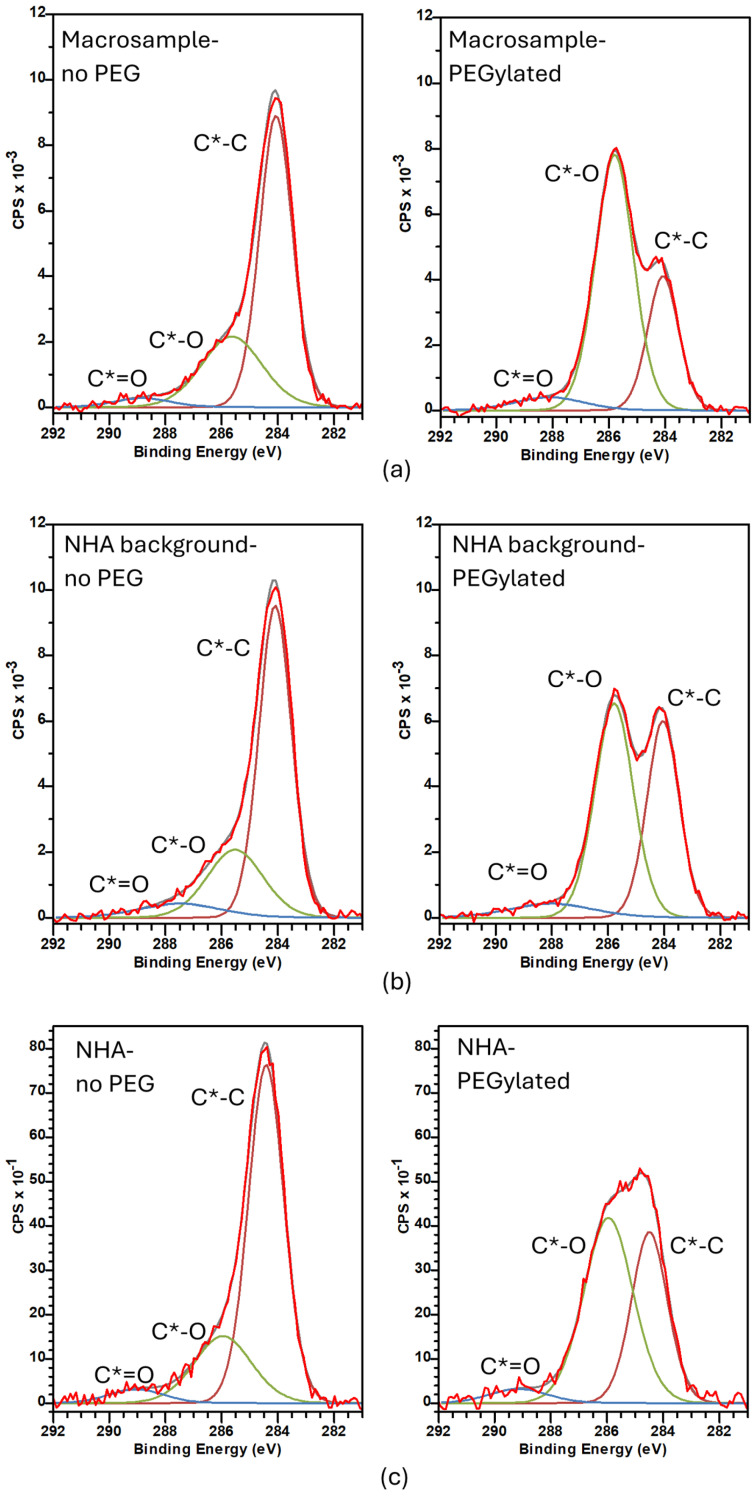
C 1s XPS spectra showing C*=O, C*-O and C*-C peaks in non-PEGylated (**left**) and PEGylated (**right**) samples for (**a**) macrosample, (**b**) NHA control/background area, and (**c**) NHA areas (measured with a smaller analysis spot size).

XPS was used to characterize the surfaces of both NHA sectors and the Au coated substrate (no nanoholes, acting as a control) on the sensor platform, as well as Au coated macrosamples. This selection of substrates was chosen to examine potential signal differences for treatments across different structures, particularly the NHA sectors with open holes. After functionalization with the PEG layer, the carbon 1s (C 1s) feature ratios relevant to the presence of a PEG layer were monitored. The XPS results indicate that the macrosample and NHA gold surfaces were successfully modified by non-homogeneous PEG self-assembly. For all sensor surfaces, the C 1s carbon peak at ≈286 eV due to C bonded to O (C*-O) increased after PEG functionalization, as shown in Figure 3a–c. The asterisk on C* denotes which carbon is releasing the detected electron. The area ratio of the C*-O peak to the C*-C peak increased for the macrosample, the NHA control, and on the NHA area after PEG functionalization, confirming the presence of PEG. The ratios are given in Table 2. C*-O/C*-C ratios increased after the PEGylation from 0.44 to 2.33 for a macrosample, from 0.37 to 1.29 for a NHA control off the sensor sector area, and from 0.32 to 1.48 for the NHA. Due to the high sensitivity of XPS, adventitious carbon can be found on all surfaces exposed to air, and the non-PEGylated spectra in Figure 3 are typical for the presence of adventitious carbon. Note that the C*-C peak detected in the PEGylated spectra in Figure 3 contains a signal from the adventitious C layer, which can show variations from sample to sample, where the PEG layer does not fully obscure the substrate signals. It is likely that minor changes in coverage affect the consistency of the ratios in Table 2.

#### 3.1.2. BSA Attachment on the NHA-Based Sensor Platforms

**Figure 4:**
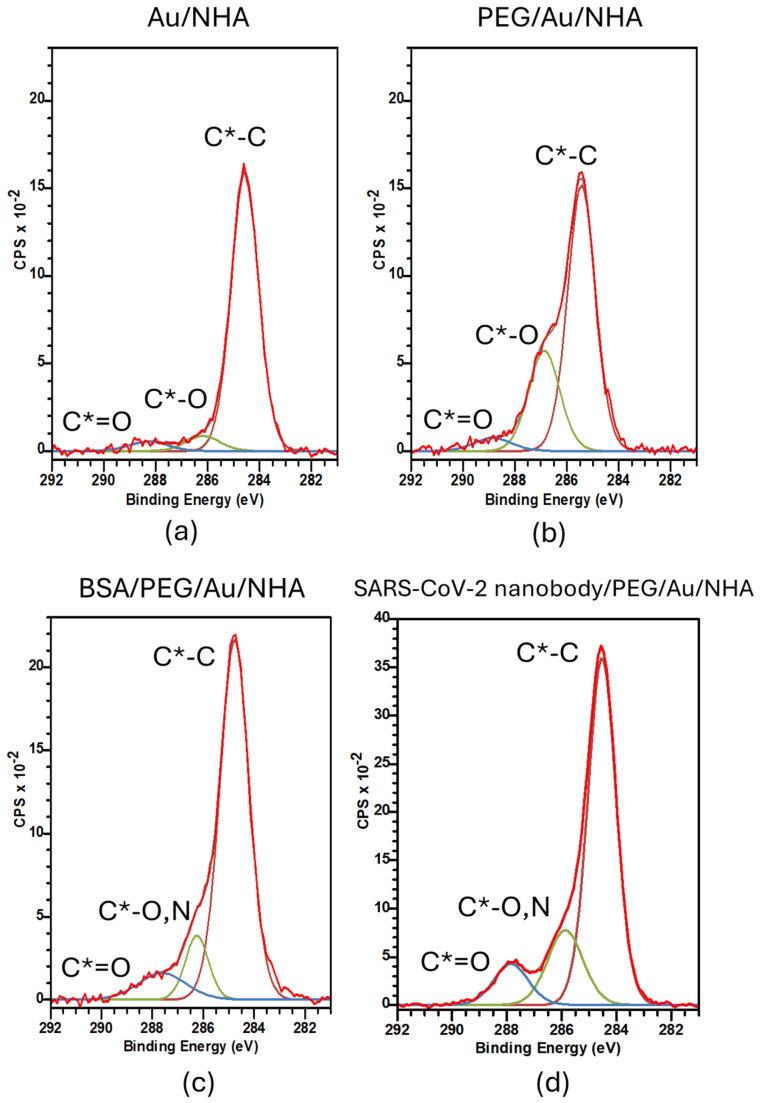
C 1s XPS spectra showing C*=O, C*-O,N and C*-C peaks for (**a**) Au/NHA, (**b**) PEG/Au/NHA, (**c**) BSA/PEG/Au/NHA, and (**d**) SARS-CoV-2 nanobody/PEG/Au/NHA samples.

BSA attachment on the surface of the NHAs was also supported by XPS analysis. High-resolution spectra for the Au 4f, C 1s, O 1s and N 1s core levels were acquired from Au/NHA, PEG/Au/NHA and BSA/PEG/Au/NHA sequence of samples. C 1s spectra for these samples are shown in Figure 4a–c. Following PEGylation, the C*-O peak increases, as in Figure 3. With BSA attachment, a significantly increased C*-C contribution is seen along with the C*-O (which is co-incident in position and shape with a possible C*-N peak) and a C*=O feature becomes more clear, consistent with the presence of a protein.

**Figure 5:**
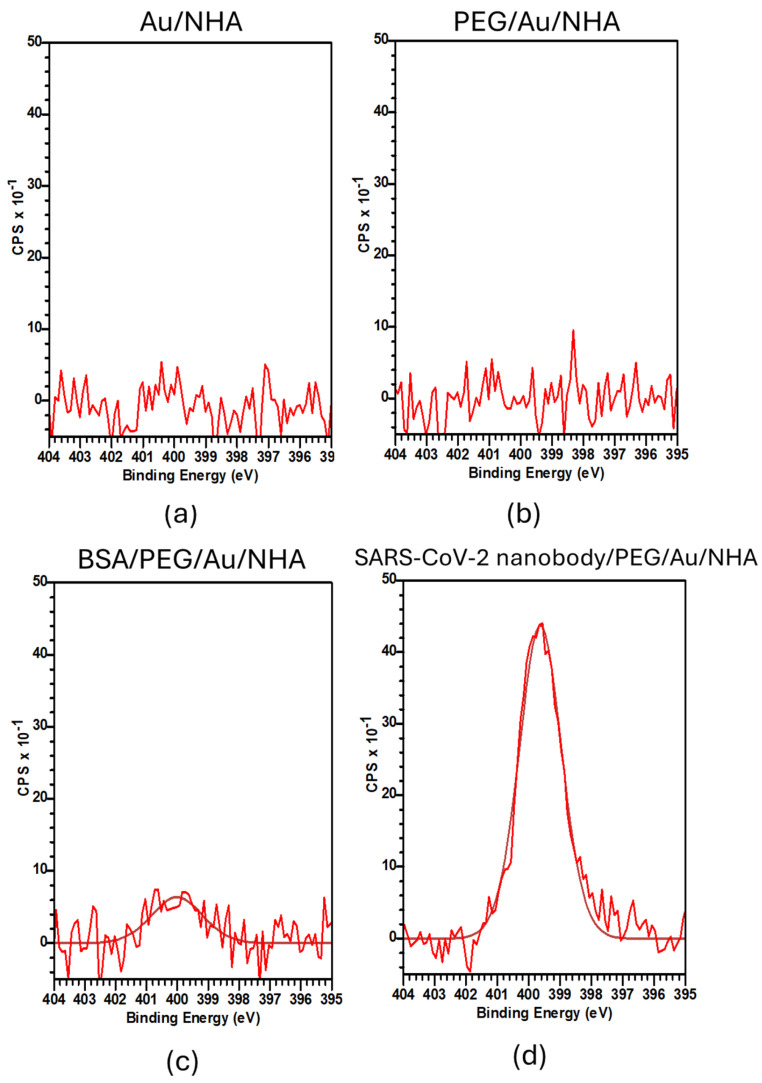
N 1s XPS spectra for (**a**) Au/NHA, (**b**) PEG/Au/NHA, (**c**) BSA/PEG/Au/NHA, and (**d**) SARS-CoV-2 nanobody/PEG/Au/NHA samples.

Similarly, N 1s spectra are shown in Figure 5a–c for Au/NHA, PEG/Au/NHA and BSA/PEG/Au/NHA samples. The presence of nitrogen is not expected on the Au/NHA and PEG/Au/NHA samples and was not observed for either of those samples. For the BSA/PEG/Au/NHA sample, however, a small nitrogen signal is observed on the NHAs, which is consistent with the presence of the bound BSA protein.

#### 3.1.3. SARS-CoV-2 Nanobody Immobilization on the NHA-Based Sensor Platforms

High-resolution XPS spectra for the SARS-CoV-2 nanobody-treated sample were acquired, and the C 1s spectrum for SARS-CoV-2 nanobody/PEG/Au/NHA samples is shown in Figure 4d. The expected trend in the C 1s peak ratios is observed in the spectrum. With nanobody attachment, both C*-O/N and C*=O features are observed, with similar but even stronger intensity than was observed for the BSA attachment.

N 1s spectra, as shown in Figure 5d, for SARS-CoV-2 nanobody/PEG/Au/NHA samples, show the strong presence of nitrogen, which is consistent with the presence of the nitrogen-containing nanobody. Also, the relative strength of the N 1s signal in Figure 5d, compared to Figure 5a–c, validates the expectation that the nanobody, a smaller protein, covers the PEGylated surface more efficiently than the larger BSA protein, which could be sterically hindered from filling the surface.

**Figure 6:**
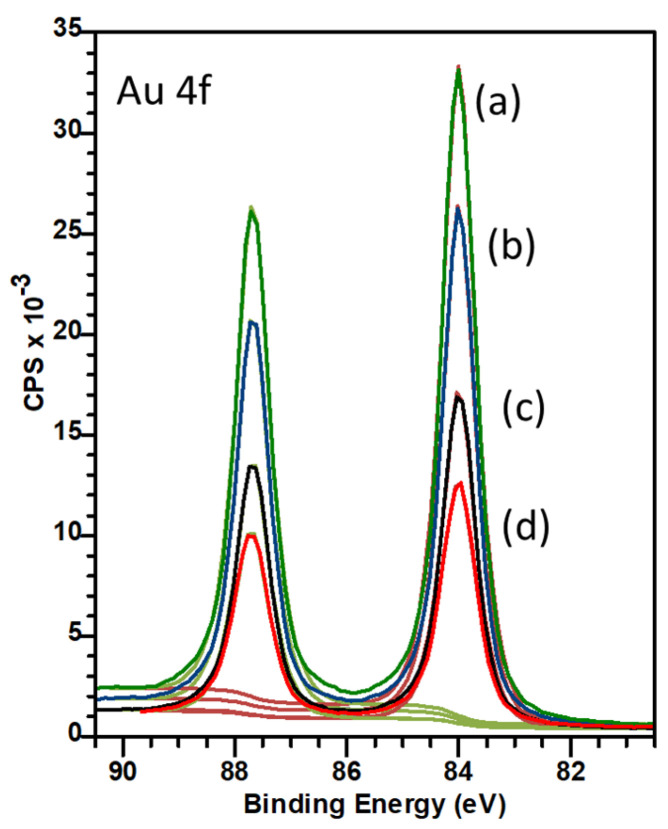
Au 4f XPS spectra for (a) Au/NHA, (b) PEG/Au/NHA, (c) BSA/PEG/Au/NHA and (d) SARS-CoV-2 nanobody/PEG/Au/NHA samples. Note Shirley background fits are shown.

The substrate Au 4f peak is expected to decrease as it is attenuated by an increasing thickness of deposition layers on the Au surface of the NHA. Thus, as seen in Figure 6, the Au peak is highest on the nonfunctionalized bare Au surface, decreases with PEGylation, and decreases further with the protein binding (BSA or nanobody). Note that the Au 4f attenuation is greatest after the SARS-CoV-2 nanobody attaches to the PEGylated surface (compared with BSA binding). These XPS observations are consistent with the binding of PEG and the two proteins onto the Au NHA surfaces. The high Au attenuation observed on the SARS-CoV-2 nanobody sample supports the relatively dense coverage of nanobodies onto the sensor PEG layer that was suggested by the large N 1s signal (Figure 5d).

### 3.2. Optical Spectroscopy Results

As noted in Section 2.2 above, quantitative analysis of the NHA-based plasmonic sensing platform sensitivity was first established using water–ethanol solutions with different concentrations and varying refractive indices (1.3330 to 1.3655) [30], which produced red shifts in the resonance peak from 615.3 ± 1.9 nm to 628.5 ± 1.0 nm [29]. From the indicated NHA platform sensitivity of 425 ± 46 nm/RIU, we specifically sought to validate surface chemistry and optical responses for functionalization procedures in sensor development. This sensitivity falls withing the range reported for recent plasmonic sensing platforms, indicating comparable performance [12,13]. (We note, therefore, that analytical assays via concentration-dependent target binding studies to determine sensor limit of detection (LOD) was beyond the scope of the current study.)

#### 3.2.1. Surface Functionalization via PEG Self-Assembly

**Figure 7:**
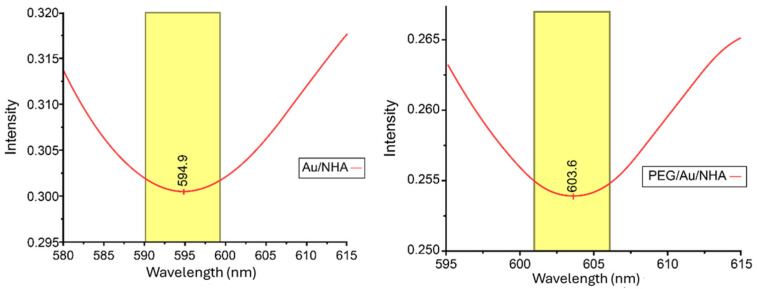
Normalized and fitted LSPR spectral measurement of (**left**) Au/NHA in PBS, and (**right**) PEG/Au/NHA in PBS.

As a foundation to LSPR sensing measurements, Au/NHA and PEG/Au/NHA samples within the microfluidic PDMS channel were also characterized with the optical spectrometer to observe their relative resonance peak positions. These experiments were performed in the PBS solution environment. The data were normalized and fitted for peak position investigation, as shown in Figure 7. The yellow region indicates the area where the peak position was searched. Both data belong to the same NHA sector before and after PEGylation. The average peak position shifted 8.7 nm with PEGylation, from 594.9 nm to 603.6 nm. This shift further indicates, in agreement with the XPS results above, that PEGylation has occurred and produces an LSPR red shift.

#### 3.2.2. BSA Attachment to the NHA-Based Sensor Platforms

**Figure 8:**
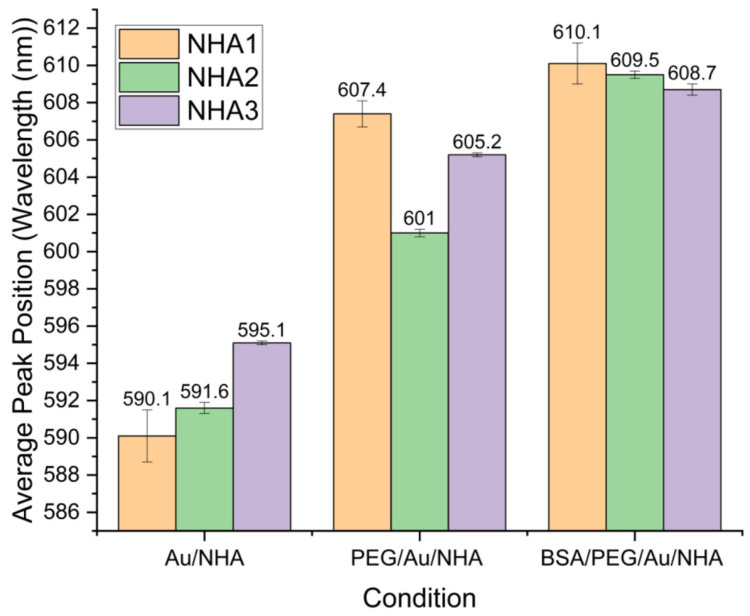
Mean peak positions and standard deviations measured at NHA1, NHA2 and NHA3 for Au/NHA, PEG/Au/NHA and BSA/PEG/Au/NHA sample conditions.

Optical spectrometer measurements were performed on three separate sectors within the NHA platform, acquiring data in three trials for each of the Au/NHA, PEG/Au/NHA and BSA/PEG/Au/NHA samples, which were all in PDMS wells with PBS solution. Table S2 and Figure 8 show that the average peak positions increase sequentially with PEGylation and then BSA attachment, as expected. The average resonance peaks for NHA1 (Sector 1 on the multi-sector NHA platform), NHA2, and NHA3 show trending red shifts for the conditions of Au/NHA, PEG/Au/NHA and BSA/PEG/Au/NHA, respectively.

Though LSPR sensing is typically a differential spectral shift technique, it is interesting to consider the average trends from these studies. The averaged peak positions using all three NHA sectors and all three trials at each are (592.3 ± 2.2) nm, (604.5 ± 2.7) and (609.5 ± 0.9) nm for the Au/NHA, PEG/Au/NHA and BSA/PEG/Au/NHA sample conditions, respectively, as shown in Table S3 and Figure S4.

For PEG attachment, the cumulative shift in the averaged peak is (12.3 ± 3.6) nm, and a (17.2 ± 2.8) nm shift (from the bare Au surface) is obtained for PEGylated plus BSA samples, as shown in Table 3. The mean peak position increases as expected, and the standard deviations offer an indication of the reproducibility of the data for these measurements.

**Figure 9:**
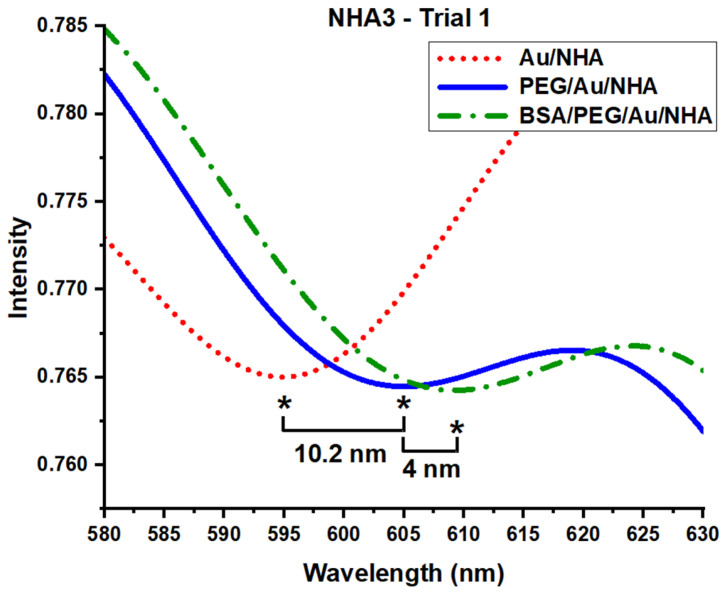
Normalized and fitted spectral measurements for Au/NHA, PEG/Au/NHA and BSA/PEG/Au/NHA sensor conditions in PBS (NHA3-Trial1) (The stars are at the minima of the various curves).

Having characterized the stepwise surface biochemistry by XPS and the associated optical property changes with LSPR (for a range of sensors and conditions) we now provide a specific example of differential-shift sensing. From the three sensor sectors and three trials on each, one set of LSPR spectral results (for NHA3-Trial 1) was selected and is shown in Figure 9. For this specific case, the resonance peaks are 594.9 nm, 605.1 nm, and 609.1 nm for the Au/NHA, PEG/Au/NHA and BSA/PEG/Au/NHA conditions, respectively. Thus, compared to the pre-PEG functionalization, the resonance peak shift is 10.2 nm after PEGylation, and 14.2 nm after subsequent BSA attachment.

It is noteworthy that, when looking at different sectors and trials, the bare NHA resonance peak in PBS exhibits a considerable level of consistency, which is indicative of the repeatability of both the sensor and the measurement setup. Similarly, the PEGylation process yielded resonance peak shifts that were quite comparable. With the subsequent BSA attachment, the average peak wavelength position increased for all samples, indicating the red shift in the resonance peak because of the increase in refractive index at the surface, which confirms successful binding. These spectrometer results for the model protein BSA are consistent with XPS findings, which indicated BSA capture onto the sensor surfaces.

#### 3.2.3. SARS-CoV-2 Nanobody Immobilization on the NHA-Based Sensor Platforms

**Figure 10:**
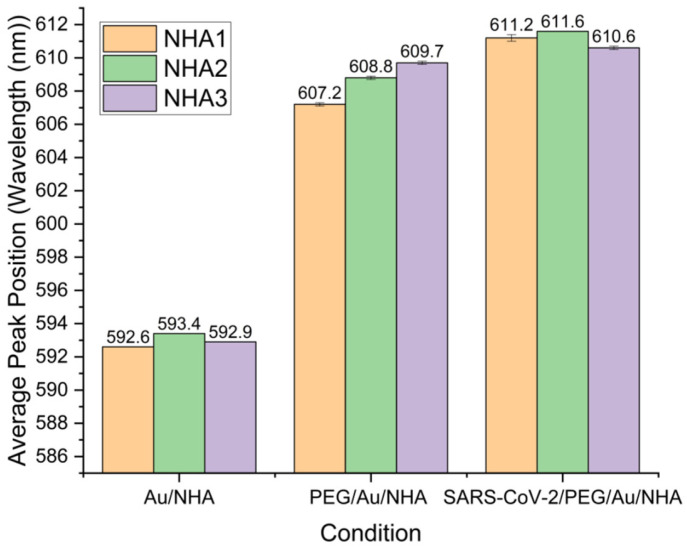
Peak positions and (small) standard deviations of NHA1, NHA2 and NHA3 for Au/NHA, PEG/Au/NHA and SARS-CoV-2 nanobody/PEG/Au/NHA conditions.

LSPR measurements were made on three sectors within the NHA platform, acquiring data in three trials each for Au/NHA, PEG/Au/NHA and SARS-CoV-2 nanobody/PEG/Au/NHA samples, which were all in a PDMS well with PBS solution. Table S4 and Figure 10 show the peak positions shift to longer wavelength with PEGylation and then SARS-CoV-2 nanobody attachment, as expected. The average resonance peaks for NHA1, NHA2, and NHA3 also reveal red shifts for the Au/NHA, PEG/Au/NHA and SARS-CoV-2 nanobody/PEG/Au/NHA conditions, respectively.

The average peak positions for all three NHA sectors and the three data trials acquired on each are (593.0 ± 0.3) nm, (608.6 ± 1.0), and (611.1 ± 0.4) nm for Au/NHA, PEG/Au/NHA and SARS-CoV-2 nanobody/PEG/Au/NHA conditions, respectively, as shown in Table S5 and Figure S5.

The observed shifts (from the bare Au surface) for the average peak positions are (15.6 ± 0.9) nm and (18.2 ± 0.4) nm for PEGylated and SARS-CoV-2 nanobody samples, respectively, as shown in Table 4. The shifts in the peak positions increase as expected, and the standard deviations are an indication of the reproducibility of the data.

**Figure 11:**
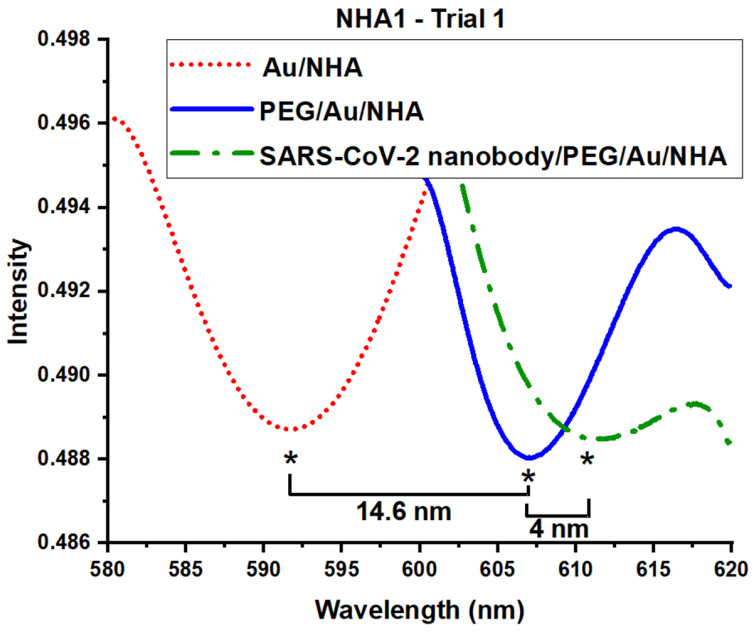
Normalized and fitted spectral measurement results for Au/NHA, PEG/Au/NHA and SARS-CoV-2 nanobody/PEG/Au/NHA sensor conditions in PBS (NHA1-Trial1) (The stars are at the minima of the various curves).

One of the measurements (NHA1-Trial 1) was chosen from three samples and three trials as an example, with its associated data shown in Figure 11. For the specific sample given, the resonance peaks are 592.6 nm, 607.2 nm, and 611.2 nm for Au/NHA, PEG/Au/NHA and SARS-CoV-2 nanobody/PEG/Au/NHA sensor platforms, respectively. Accordingly, the cumulative shift in resonance peak is 14.6 nm after PEGylation, and 18.6 nm after SARS-CoV-2 nanobody attachment.

XPS and LSPR results have been shown to be complementary to each other in this study. Both techniques show the successful binding of the layers PEG, BSA and SARS-CoV-2 nanobody. XPS further verified the chemical composition present for the different cases. While the XPS and LSPR do not report directly on the bonding mechanism, we speculate that the proteins (BSA, nanobodies) interact directly with the carboxylic acid functional group of the PEGs.

BSA served as our model protein to initially test and refine the functionalization protocol before moving to SARS-CoV-2 nanobody attachment. The nanobody receptor binding was confirmed through consistent resonance peak shifts as well as by XPS chemical analysis, to establish the sensor’s potential for biomedical pathogen detection. This study reveals the NHA-based sensor platform can support reproducible surface functionalization and specific receptor immobilization in liquid-phase conditions. This sensor platform will enable future sensing applications with additional antigen binding, specificity panel, and analytical performance evaluation studies.

## 4. Conclusions

A gold-coated nanohole array (NHA) platform has been successfully developed and validated as a localized surface plasmon resonance (LSPR) biosensor for liquid-phase, label-free detection of biomolecular interactions. The sensor system described here integrates optimized gold nanostructures with a PDMS microfluidic system to enable near-real-time measurements relevant for biomedical diagnostics. The optical spectroscopy and XPS results support the conclusion that SAM with PEGylation and subsequent specific receptor immobilization were successfully achieved on the sensor surfaces. The measured wavelength shifts were consistent with refractive index changes expected from each surface functionalization step. This study is intended as careful surface functionalization validation rather than a full analytical sensing performance evaluation. Therefore, further systematic investigations for target protein detection would proceed from the type of basis reported here.

Surface functionalization via PEGylation self-assembly was crucial for subsequent attachment of a model protein and nanobody receptor. LSPR measurements demonstrated a reproducible positive shift in peak position with each additional surface functionalization. To further confirm that LSPR responses to stepwise interfacial treatments originated from expected binding events, those processes were separately characterized by XPS measurements which monitored changes in carbon, nitrogen and gold core-level spectral features. This approach provided confirmation of each step in the sensor development toward target detection. We note also that many experiments were performed, with multiple trials carried out on three separate NHA sectors (within a multi-sector NHA platform) to provide important statistical information for assessing sensing reproducibility.

## Figures and Tables

**Table 1:** Names for various samples studied.

Abbreviation	Sample
Au/NHA	Au coated NHA-based sensor platform
PEG/Au/NHA	PEGylated Au coated NHA-based sensor platform
BSA/PEG/Au/NHA	BSA immobilized, PEGylated Au coated NHA-based sensor platform
SARS-CoV-2 nanobody/PEG/Au/NHA	SARS-CoV-2 nanobody immobilized, PEGylated Au coated NHA-based sensor platform
Macrosample	Au coated bare Si wafer without NHA
NHA background	NHA sample focused on the area outside NHA sectors

**Table 2:** C*-O/C*-C ratios for non-PEGylated and PEGylated samples.

C*-O/C*-C	Non-PEGylated	PEGylated
Macrosample	0.44	2.33
NHA background	0.37	1.29
NHA	0.32	1.48

**Table 3:** Shifts in the average peak positions and standard deviations of PEG/Au/NHA and BSA/PEG/Au/NHA conditions.

Condition	Shift in the Average Peak Position (nm)	Standard Deviations (nm)
PEG/Au/NHA	12.3	3.6
BSA/PEG/Au/NHA	17.2	2.8

**Table 4:** Peak positions and standard deviations of NHA1, NHA2 and NHA3 for PEG/Au/NHA and SARS-CoV-2 nanobody/PEG/Au/NHA conditions.

Condition	Shift in the Peak Position (nm)	Standard Deviations (nm)
PEG/Au/NHA	15.6	0.9
SARS-CoV-2 nanobody/PEG/Au/NHA	18.2	0.4

## Data Availability

The data presented in this study are available in the article.

## Supplementary Materials

The following supporting information can be downloaded at: https://www.mdpi.com/article/10.3390/s26113434/s1. Figure S1: Linear model fit to data using reported CRC refractive indices (data weighted using standard deviations), slope = 425 ± 46 nm/RIU; Figure S2. Surface modification chemistry of PEGylation.; Figure S3. Schematic of PEG solution mixing and SAM PEGylation; Figure S4. Average peak positions and standard deviations for Au/NHA, PEG/Au/NHA and BSA/PEG/Au/NHA conditions; Figure S5. Average peak positions and standard deviations for Au/NHA, PEG/Au/NHA and SARS-CoV-2 nanobody/PEG/Au/NHA conditions; Table S1: Values for the average resonance peak position in wavelength (nm) reported from our previous measurements [29] at the refractive index of different water/ethanol solution samples [30]; Table S2. Peak positions and standard deviations measured at NHA1, NHA2 and NHA3 for Au/NHA, PEG/Au/NHA and BSA/PEG/Au/NHA sample conditions; Table S3. Average peak positions and standard deviations for three trials on three cursors of Au/NHA, PEG/Au/NHA and BSA/PEG/Au/NHA samples; Table S4. Peak positions and standard deviations of NHA1, NHA2 and NHA3 for Au/NHA, PEG/Au/NHA and SARS-CoV-2 nanobody/PEG/Au/NHA conditions; Table S5. Average peak positions and standard deviation for three trials on three cursors of Au/NHA, PEG/Au/NHA and SARS-CoV-2 nanobody/PEG/Au/NHA samples.
