# Supplementary material for: Surface Functionalization Studies in the Development of Nanohole Plasmonic Sensors
Source: Sensors (Basel). 2026 May 29;26(11):3434. doi: 10.3390/s26113434 (PMC13259320; doi:10.3390/s26113434)
Supplement: Supplementary file 1 [file sensors-26-03434-s001.zip › sensors-4162949-supplementary.pdf]

Supplementary Materials

Surface Functionalization Studies in the Development of Nanohole Plasmonic Sensors

Sezin Sayin [1,2,*], Kristen L. Steffens [2], Kurt D. Benkstein [2], Mona Zaghloul [1] and Steve Semancik [2]

[1] Department of Electrical and Computer Engineering, School of Engineering and Applied Science, The George Washington University, Washington, DC 20052, USA; zaghloul@gwu.edu
[2] Biomolecular Measurement Division, National Institute of Standards and Technology (NIST), Gaithersburg, MD 20899, USA; kristen.steffens@nist.gov (K.L.S.); kurt.benkstein@nist.gov (K.D.B.); stephen.semancik@nist.gov (S.S.)
* Correspondence: sezinsayin@gwu.edu

Table S1. Values for the average resonance peak position in wavelength (nm) reported from our previous measurements [1] at the refractive index of different water/ethanol solution samples [2]. A linear fit to these data produces a platform sensitivity of 425 ± 46 nm/RIU (Igor Pro 9.05). Water and ethanol densities at 20 °C are 0.998207 g/cm^3 and 0.7893 g/cm^3, respectively [2].

Average Resonance Peak Position (nm)	Ethanol Volume Ratio	Ethanol Mass Ratio	Refractive Index CRC [2]
615.3±1.9	0	0	1.3330
619.6±0.9	0.2	0.17	1.3447
624.0±1.0	0.4	0.35	1.3562
627.5±0.7	0.6	0.54	1.3626
628.5±1.0	0.8	0.76	1.3655

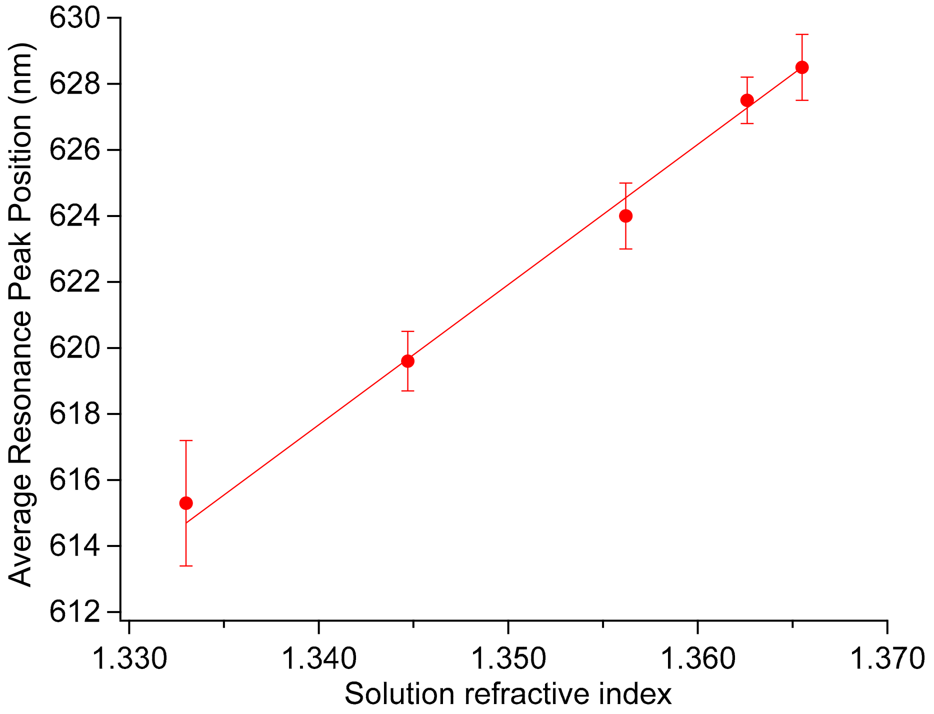

Figure S1. Linear model fit to data using reported CRC refractive indices (data weighted using standard deviations), slope = 425 ± 46 nm/RIU.

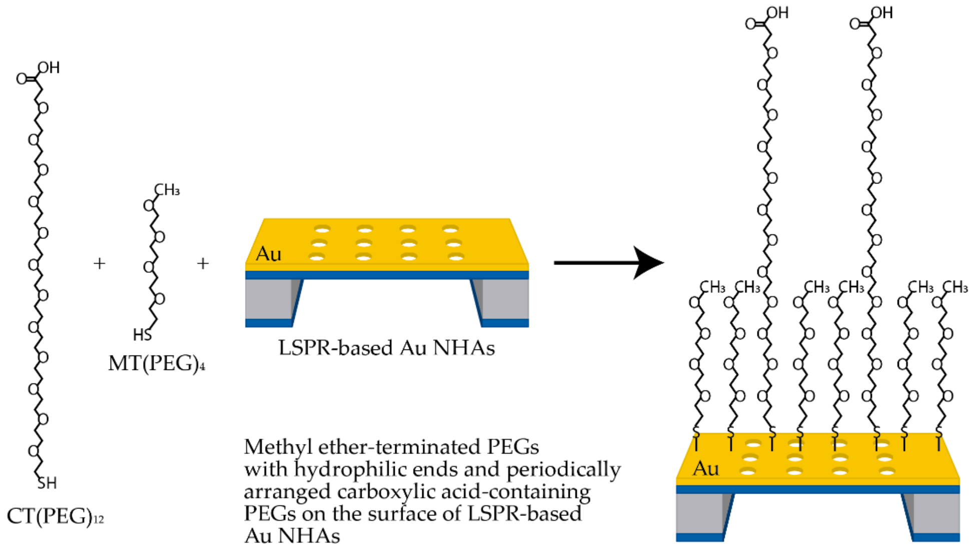

Figure S2. Surface modification chemistry of PEGylation.

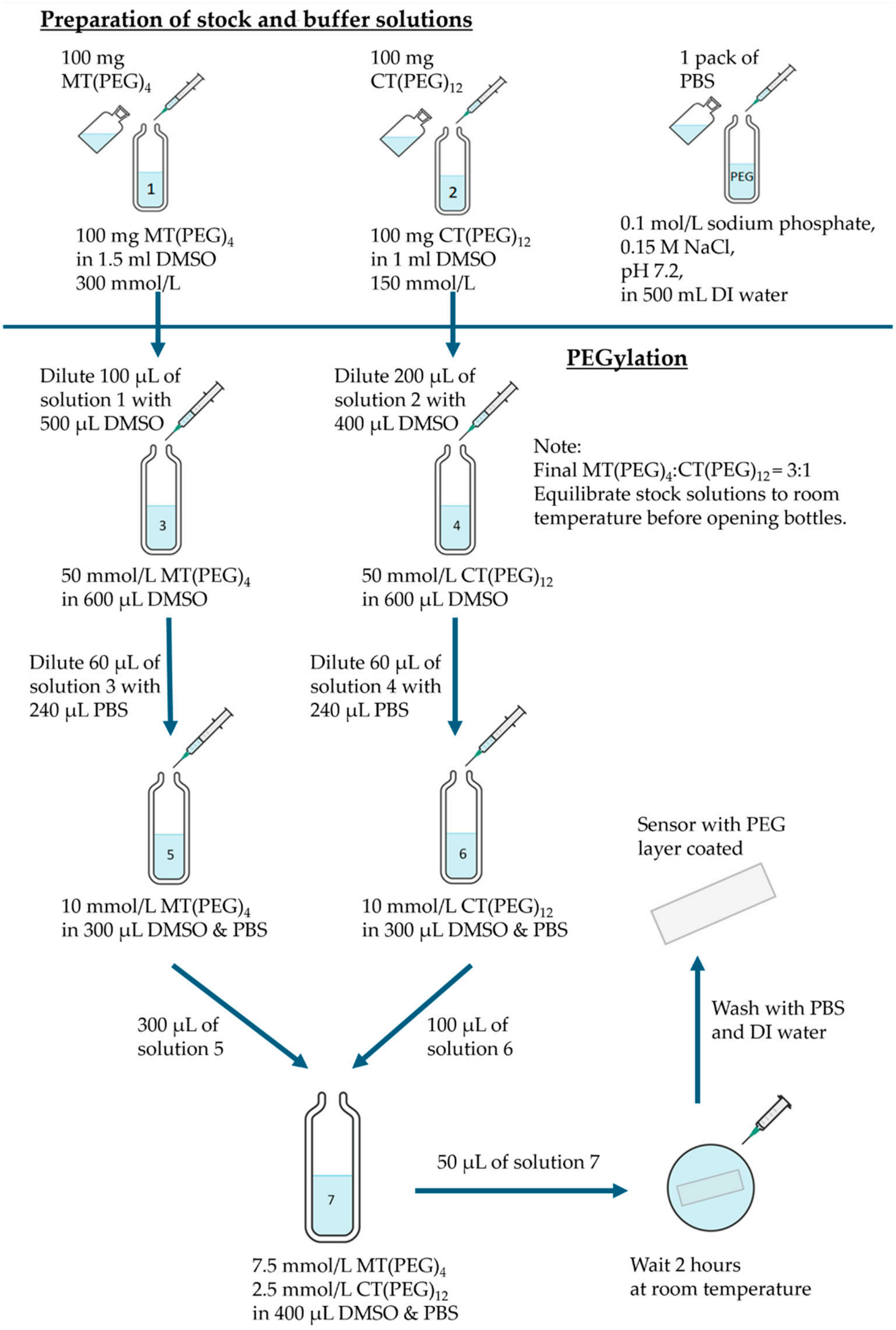

Figure S3. Schematic of PEG solution mixing and SAM PEGylation.

Reported mean peak positions and standard deviations in the following tables and figures are all based upon $n = 3$ measurements.

Table S2. Peak positions and standard deviations measured at NHA1, NHA2 and NHA3 for Au/NHA, PEG/Au/NHA and BSA/PEG/Au/NHA sample conditions.

Condition	NHA1 (nm)	NHA2 (nm)	NHA3 (nm)
Au/NHA	590.1±1.4	591.6±0.3	595.1±0.1
PEG/Au/NHA	607.4±0.7	601.0±0.2	605.2±0.1
BSA/PEG/Au/NHA	610.1±1.1	609.5±0.2	608.7±0.3

Table S3. Average peak positions and standard deviations for three trials on three cursors of Au/NHA, PEG/Au/NHA and BSA/PEG/Au/NHA samples.

Condition	Average Peak Position (nm)	Standard deviations (nm)
Au/NHA	592.3	2.2
PEG/Au/NHA	604.5	2.7
BSA/PEG/Au/NHA	609.5	0.9

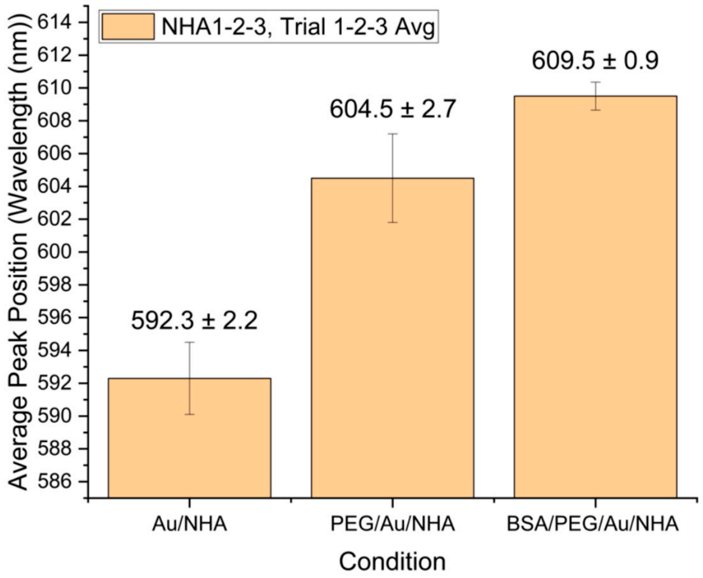

Figure S4. Average peak positions and standard deviations for Au/NHA, PEG/Au/NHA and BSA/PEG/Au/NHA conditions.

Table S4. Peak positions and standard deviations of NHA1, NHA2 and NHA3 for Au/NHA, PEG/Au/NHA and SARS-CoV-2 nanobody /PEG/Au/NHA conditions.

Condition	NHA1 (nm)	NHA2 (nm)	NHA3 (nm)
Au/NHA	592.6±0	593.4±0	592.9±0
PEG/Au/NHA	607.2±0.1	608.8±0.1	609.7±0.1
SARS-CoV-2 nanobody/PEG/Au/NHA	611.2±0.2	611.6±0	610.6±0.1

Table S5. Average peak positions and standard deviation for three trials on three cursors of Au/NHA, PEG/Au/NHA and SARS-CoV-2 nanobody/PEG/Au/NHA samples.

Condition	Average Peak Position (nm)	Standard deviations (nm)
Au/NHA	593.0	0.3

PEG/Au/NHA	608.6	1.0
SARS-CoV-2 nanobody/ PEG/Au/NHA	611.1	0.4

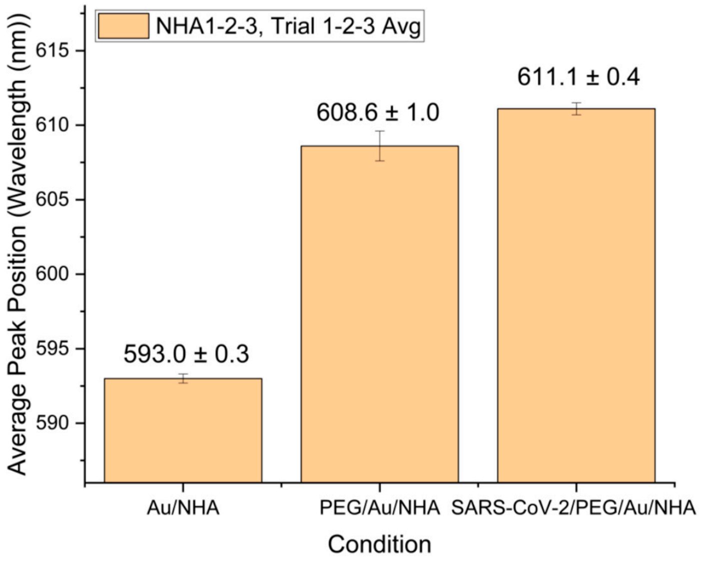

Figure S5. Average peak positions and standard deviations for Au/NHA, PEG/Au/NHA and SARS-CoV-2 nanobody/PEG/Au/NHA conditions.

NIST Equipment/Supplies Disclaimer: Commercial equipment and materials are identified to adequately specify certain procedures. In no case does such identification imply recommendation or endorsement by the National Institute of Standards and Technology, nor does it imply that the materials or equipment identified are necessarily the best available for the purpose.

References

1. Sayin, S., Zhou, Y., Wang, S., Acosta Rodriguez, A., & Zaghloul, M. (2023). Development of liquid-phase plasmonic sensor platforms for prospective biomedical applications. Sensors, 24(1), 186.
2. Rumble, J. R. (Ed.). (2025). CRC handbook of chemistry and physics (106th ed.). CRC Press: Boca Raton, FL, USA, 2025.
